# Real-World Assessment of Weight Change in People with HIV-1 After Initiating Integrase Strand Transfer Inhibitors or Protease Inhibitors

**DOI:** 10.36469/jheor.2020.13457

**Published:** 2020-07-16

**Authors:** Yen-Wen Chen, Helene Hardy, Christopher D. Pericone, Wing Chow

**Affiliations:** 1 Janssen Scientific Affairs, LLC, Titusville, NJ, USA; 2 Janssen Research & Development, LLC, Titusville, NJ, USA

**Keywords:** weight gain, HIV, integrase inhibitors, antiretroviral therapy, women

## Abstract

**Background:**

Studies have shown an increase in weight among people living with human immunodeficiency virus (PLWH) who have also initiated integrase strand transfer inhibitors (INSTI). However, limited data are available regarding comparison of these changes with other antiretroviral regimens.

**Objective:**

To assess differences in weight gain after initiating INSTI- versus protease inhibitor (PI)-based regimens among treatment-naïve PLWH overall, and among a subpopulation of females only.

**Methods:**

This retrospective, observational cohort study included data from the Optum ^®^ deidentified Electronic Health Record (EHR) database. Adult PLWH who initiated INSTI- or PI-based regimens between March 1, 2016 and June 30, 2018 (index date was the first INSTI or PI prescription in this period) with ≥12-month baseline and follow-up periods, ≥1 weight measure during each period, and no prior antiretroviral use were included. The last weight measure between 12 months pre- and 30 days post-index was defined as baseline weight; the last measure between the months 4 and 12 of follow-up was defined as post-weight. Weight change was reported as absolute change and proportion of patients with increased weight. Cohorts were balanced using propensity score (PS) matching. Multivariable models were used to compare outcomes of interest.

**Results:**

After matching, 1588 patients were included (794 per cohort). At baseline, 46% were <50 years old, 26% were females, 12% had Type II diabetes and 30% had hypertension (mean baseline weight: INSTI: 83 kg (183 lb), PI: 82 kg (181 lb); *P* = 0.3). The mean time to follow-up weight measure was 9.3 months; INSTI initiators had a 1.3 kg (2.9 lb) greater mean weight gain (95% CI: 0.5–2.0), and a higher proportion with ≥5% weight gain (30.7% vs 26.1%; [OR=1.3, 95% CI: 1.0–1.6]) than PI initiators. Differences in weight gain between regimens were larger among females; female INSTI initiators had a 2.5 kg (5.3 lb) greater mean weight gain (95% CI: 0.7–4.2) and a higher proportion with ≥5% weight gain (37.5% vs 26.4%; OR=1.7; 95% CI [1.1–2.6]) than PI initiators.

**Conclusion:**

In a real-world setting, compared to PI-based regimens, INSTI-based regimens are associated with greater weight gain for treatment-naïve PLWH. This study may inform HIV treatment choice for health care providers.

## INTRODUCTION

In the United States, human immunodeficiency virus (HIV) accounts for >16 000 deaths per year and a substantial burden on the >1.1 million people living with HIV (PLWH). 1,2 Improved antiretroviral (ARV) therapies have increased the life expectancy of PLWH, 3 increasing the proportion of PLWH ≥50 years old from 42% to 50% from 2013 to 2016. 2016.^4^ Elderly PLWH are at greater risk of developing chronic diseases such as hypertension and diabetes, and these conditions are increasingly prevalent in PLWH. 5–7 Furthermore, metabolic disease risk factors such as obesity may interact with HIV or ARV-related factors, thereby worsening existing comorbidities and/or complicating patient care. 8

Recent studies have shown that initiating ARV therapy leads to weight gain, from a few kilograms to >20% of original bodyweight, especially within the first year.^9^–^11^ Integrase strand transfer inhibitor (INSTI)-based regimens are commonly prescribed for the management of HIV^12^ but have recently been associated with greater weight gain compared with protease inhibitor (PI)-based^13^ or non-nucleoside reverse transcriptase inhibitor (NNRTI)-based regimens.^14^,^15^ Impacts on weight vary, with dolutegravir and bictegravir demonstrating greater weight gain than other INSTIs.^14^,^16^ Recent evidence also suggests greater risk of weight gain in females using INSTI-based regimens compared with males;^17^ however, few studies have compared weight gain with INSTI-based regimens versus other regimens in females.^18^ Therefore, the current study aimed to compare weight gain among treatment-naïve PLWH who initiated INSTI-based versus PI-based ARV regimens, including the subpopulation of female patients.

## METHODS

### Data Source(s)

Patient-level records were obtained from the Optum^®^ deidentified EHR database, containing deidentified longitudinal data for 80 million US patients (≥7 million patients from each Census region). The database includes information on outpatient visits, diagnostic procedures, medications, laboratory results, hospitalizations, clinical notes, and patient outcomes, primarily from integrated delivery networks.

### Study Period and Population

**Figure 1:**
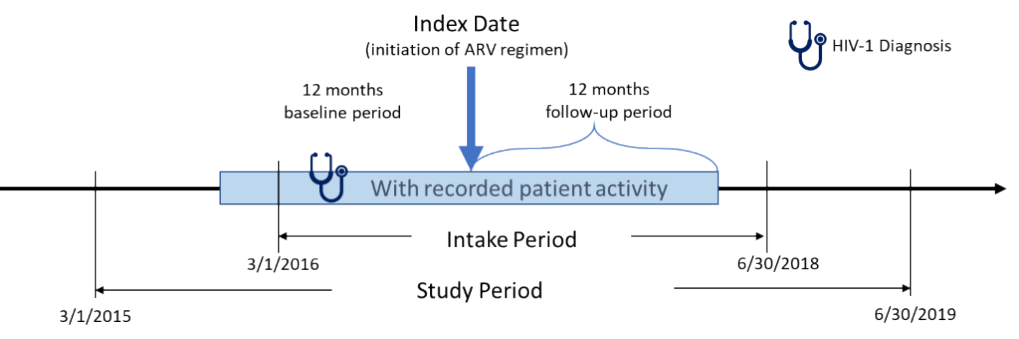
Study Design and Timeframe Abbreviations: ARV, antiretroviral; HIV, human immunodeficiency virus.

This retrospective, observational, matched-cohort study was conducted using data from March 1, 2015 to June 30, 2019 (Figure 1). PLWH who had ≥1 written prescription for INSTI- or PI-based ARV regimens between March 1, 2016 and June 30, 2018 were included. The date of the earliest written ARV prescription during this study intake period was considered the index date. Additional study inclusion criteria were: age ≥18 years at index; ≥365 days of EHR activity both pre- and post-index; ≥1 diagnosis for HIV-1 during the year prior to the index date (baseline period); ≥1 baseline (between 12 months pre- and 30 days post-index) and follow-up (between months 4 and 12 post-index) measure for either weight or body mass index (BMI). Exclusion criteria were: HIV-2 diagnosis or evidence of pregnancy during the study; ≥1 prescription for any ARV during baseline period; and inconsistent/missing data on gender or birth year.

### Identification of Index Regimen

For treatment-naïve HIV-1 patients, an ARV regimen generally consists of two nucleoside reverse transcriptase inhibitors (NRTIs) in combination with a third ARV agent from other ARV drug classes (INSTI, PI, or NNRTI). ARV regimens are currently available in two types of formulations: fixed dose combinations (FDC), which contain a combination of two or more ARV agents in a single tablet, and multi-tablet regimen formulations. For this study, INSTI- or PI-based regimens identified on the index date were defined as the index regimen. Patients were classified as INSTI initiators if the index ARV regimen included dolutegravir, raltegravir, bictegravir, or elvitegravir, with no prescription for PIs or NNRTIs within ±14 days of the index date. Patients were classified as PI initiators if the index ARV regimen included atazanavir, darunavir, fosamprenavir, ritonavir, saquinavir, tipranavir, amprenavir, indinavir, or nelfinavir, with no prescription for INSTIs or NNRTIs within ±14 days of the index date. Patients not taking an FDC INSTI or PI were required to have prescriptions for ≥2 distinct NRTIs or ≥1 NRTI FDC within 14 days of the index date.

### Outcomes

The primary outcomes were change in weight and BMI within 12 months post-initiation of INSTI- versus PI-based regimens in treatment-naïve (1) males and females and (2) females. Change in weight and BMI from baseline measure to follow-up measure were reported as absolute change and percent change. The proportion of patients with weight gain ≥10 kg, ≥5%, ≥10%, or extreme gain (i.e., weight gain above the ≥95th percentile for the overall study population) were also determined, as were the proportion with BMI increases ≥5%, ≥90th percentile or ≥95th percentile of the overall study population, or who shifted their BMI category. All outcomes were also determined by index BMI value category (<25 kg/m^2^ vs ≥25 kg/m^2^).

### Variables

Variables included demographic characteristics (age, gender, race, region, insurance type, and index year), clinical characteristics (Quan Charlson Comorbidity Index [QCI] score; comorbidities such as diabetes, hypertension, and obesity [see medication and diagnostic codes in the Supplementary Material], and medication use [see medication and diagnostic codes in the Supplementary Material]).

### Data Analysis

Demographic characteristics, clinical characteristics, and outcomes of interest were analized descriptively using univariate statistics. Frequencies and percentages were reported for categorical variables; means and SDs were reported for continuous variables. All analyses used SAS Enterprise Guide, version 7 (SAS Institute Inc., Cary, NC).

### Propensity Score Matching

Propensity score (PS) matching was applied to control for selection bias and improve cohort comparability. Logistic regression was employed to fit a PS model where the outcome was initiation of INSTI-based versus PI-based regimens. Independent variables in the model included: age, race, region, index year, weight and BMI, clinical characteristics, various comorbidities associated with weight gain (prediabetes/glucose intolerance, Type II diabetes, cardiovascular disease, myocardial infarction, peripheral vascular disease, congestive heart failure, hypertension, hyperlipidemia, obesity, nonalcoholic steatohepatitis [NASH], AIDS, and cancer), and commonly used medications that are associated with weight gain (diabetes medications, psychiatric/neurologic medications, chronic oral corticosteroids [≥2 consecutive written prescriptions for steroid with days’ supply ≥60 days during baseline period], hormone therapy/contraception, appetite stimulants/suppressants, and antihypertensives).

Each INSTI-based regimen user was matched to a PI-based regimen user with similar predicted probability using greedy nearest neighbor 1:1 matching (with no replacement), and a caliper with width of 0.2 of the pooled SD of the logit (PS) was used. Random numbers were assigned to all the patients in the INSTI-based regimen cohort using random number generation with a specified seed so that if two or more patients in the PI-based regimen cohort had the same PS and were considered the best match for a patient in the INSTI-based regimen cohort, the patient with the numerically lowest random number would be chosen as a match. Separate matching was conducted for the overall analysis and the female analysis to provide optimally balanced variables and maximum possible sample size for each.

### Outcome Models

Outcomes of interest were compared between PS-matched cohorts using multivariable regression models. Confounders not balanced by PS-matching were controlled in the multivariable models as a covariate. Ordinary least squares models were used to model absolute and expected percent change in weight and BMI, as a function of the independent variable (INSTI- vs PI-based regimens) and covariates. 95% CIs and *P* values were calculated. Logistic regression was used to model the expected proportion of patients having weight/BMI increases, as a function of the independent variable (INSTI- vs PI-regimens) and covariates. *P* < 0.05 was considered statistically significant for all analyses.

## RESULTS

### Baseline Demographic and Clinical Characteristics

Of the 28 782 patients with ≥1 prescription for an INSTI or PI-based regimen during the intake period and ≥1 HIV-1 diagnosis within the prior 12 months, 5117 patients met all inclusion and exclusion criteria (INSTI, 4306; PI, 811). After matching (see patient attributes pre/post matching in the Supplementary Material), 1588 patients remained (794 per cohort). At baseline, the mean age was 49 years, 93% were ≤65 years of age, 26% were females, 64% had an AIDS diagnosis, 12% had Type II diabetes, and 30% had hypertension. The mean baseline weight was 83 kg (183 lb) and 82 kg (181 lb) (*P* = 0.3); mean time to follow-up weight measure was 291 days and 286 days (*P* = 0.2) for the INSTI-based and PI-based cohorts, respectively (Table 1).

Among INSTI initiators, 25 (3.1%) were underweight at baseline (BMI <18.5 kg/m^2^), 269 (33.9%) had normal bodyweight (BMI 18.5–24.9 kg/m^2^), 261 (32.9%) were overweight (BMI 25.0–29.9 kg/m^2^), and 239 (30.1%) were obese (BMI ≥30 kg/m^2^). Among PI initiators, 18 (2.3%) were underweight at baseline, 304 (38.3%) had normal bodyweight, 243 (30.6%) were overweight, and 229 (28.8%) were obese (Table 1).

### Overall Weight and BMI Changes

**Figure 2:**
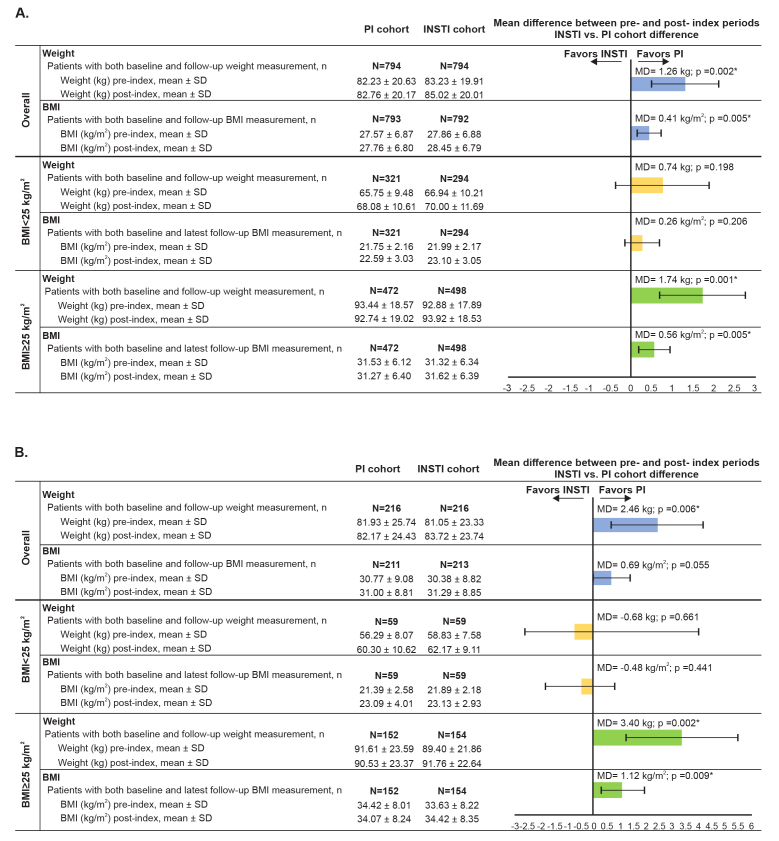
Mean Change in Weight and BMI, Overall and by Baseline BMI Abbreviations: BMI, body mass index; INSTI, integrase strand transfer inhibitor; MD, mean difference; PI, protease inhibitor; SD, standard deviation. Figure 2A indicates mean change in weight for all patients; among patients with baseline BMI ≥25 kg/m^2^, those with BMI ≥5% increases were 28.9% vs. 19.5% for INSTI and PI, respectively; OR=1.68; 95% CI [1.25–2.27]. Among patients with baseline BMI <25 kg/m^2^, those with BMI ≥5% increases were 40.1% vs. 36.1% for INSTI and PI, respectively; OR=1.19; 95% CI [0.86–1.64]. Figure 2B indicates mean change in weight for females only. For Figure 2B, the mean differences are calculated from adjusted values. * indicates *P* < 0.05.

**Figure 3:**
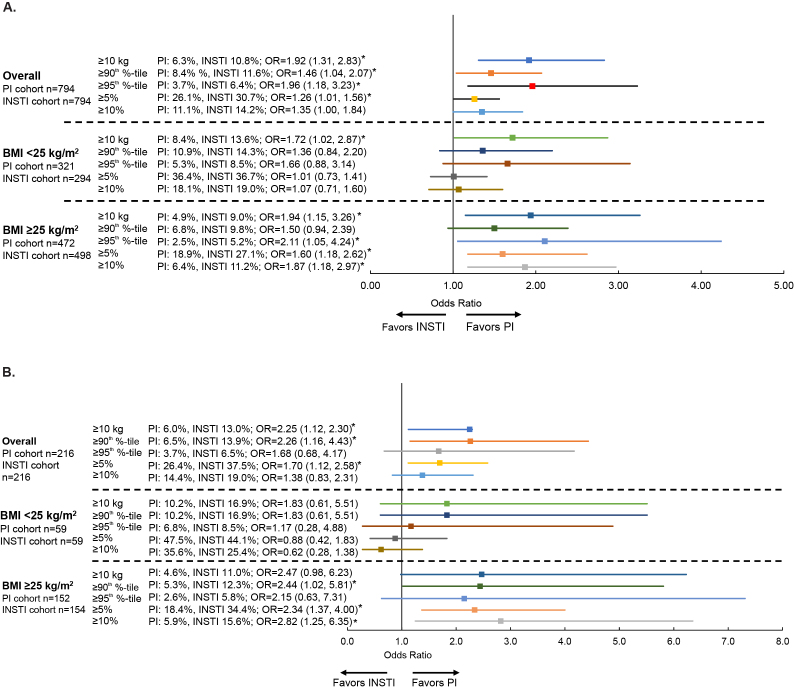
Proportion of Patients with Weight Gain, Overall and by BMI Abbreviations: BMI, body mass index; INSTI, integrase strand transfer inhibitor; OR, odds ratio; PI, protease inhibitor. Figure 3A is for all patients and Figure 3B is for females only. * indicates *P* < 0.05.

After a mean ~9.3-month follow-up, INSTI initiators had a 1.3 kg (2.9 lb) greater mean weight gain (1.8 kg [4.0 lb] vs 0.5 kg [1.1 lb]; 95% CI [0.5–2.0]) compared with PI initiators (Figure 2A). Similarly, a greater proportion of INSTI initiators experienced ≥5% (30.7% vs 26.1%; OR=1.3; 95% CI [1.0–1.6]), ≥10 kg (22 lb) weight gain (10.8% vs 6.3%; OR=1.9; 95% CI [1.3–2.8]), and extreme weight gain (i.e., weight gain ≥95th percentile for the overall study population; ≥13.6 kg [29.9 lb]; 6.4% vs 3.7%; OR=2.0; 95% CI [1.2–3.2]) (Figure 3A).

INSTI initiators had a greater mean BMI increase (0.4 kg/m^2^) compared with PI initiators and a higher proportion experienced BMI increases ≥5% (33.1% vs 26.2%; OR=1.4; 95% CI [1.1–1.7]) or ≥90th percentile (3.3 kg/m^2^) (11.7% vs 8.3%; OR=1.5; 95% CI [1.1–2.0]), compared with PI initiators.

### Overall Weight and BMI Changes by Baseline BMI

Among patients with baseline BMI ≥25 kg/m^2^, INSTI initiators experienced a 1.7 kg (3.7 lb) greater weight gain (1.0 kg [2.2 lb] vs −0.7 kg [−1.5 lb]; 95% CI [0.7–2.8)]) than PI initiators (Figure 2A). Similarly, a greater proportion of INSTI initiators with baseline BMI ≥25 kg/m^2^ experienced ≥5% weight gain (27.1% vs 18.9%; OR=1.6; 95% CI [1.2–2.6]), ≥10% weight gain (i.e., 8.3 kg [18.3 lb]; 11.2% vs 6.4%; OR=1.9; 95% CI [1.2–3.0]), ≥10 kg (22 lb) weight gain (9.0% vs 4.9%; OR=1.9; 95% CI [1.2–3.3]), or extreme weight gain (i.e., weight gain ≥95th percentile for the overall study population; 5.2% vs 2.5%; OR=2.1; 95% CI [1.1–4.2]; Figure 3A). Among patients with baseline BMI <25 kg/m^2^, differences in mean weight gain between cohorts were not statistically significant (Figure 2A), although a greater proportion of INSTI initiators experienced ≥10 kg (22 lb) weight gain (13.6% vs 8.4%; OR=1.7; 95% CI [1.0–2.9]; Figure 3A).

Among patients with baseline BMI ≥25 kg/m^2^, INSTI initiators had a significantly greater BMI increase (0.6 kg/m^2^) and a significantly greater proportion with BMI increases ≥5% (28.9% vs 19.5%; OR=1.7; 95% CI [1.3–2.3]), compared with PI initiators, but there were no statistically significant differences in BMI changes among patients with baseline BMI <25 kg/m^2^ (Figure 2A).

Among INSTI initiators with normal weight at baseline, 22.3% and 3.3% became overweight or obese, respectively. Among overweight INSTI initiators, 17.4% shifted to obese. Among normal weight PI initiators, 19.1% and 1.7% became overweight or obese, respectively. Among overweight PI initiators, 13.6% shifted to obese (Table 2).

### Baseline Demographic and Clinical Characteristics for Females

Of the 5117 patients who met all inclusion and exclusion criteria, 1212 were females. Separate matching was conducted for the female analysis. After matching, 432 patients remained (N = 216 matched pairs). Index year, baseline AIDS and steroid use remained unbalanced after matching. Thus, they were adjusted as covariates in the regression models. At baseline, the mean age was 49 years, 93% were ≤65 years of age, 16% had Type II diabetes, and 37% had hypertension. The most commonly reported race among females was African American (55%), followed by white (37%). The mean baseline weight was 81 kg (179 lb) and 82 kg (181 lb) (*P* = 0.7); mean time to follow-up weight measure was 295 days and 286 days (*P* = 0.3) for the INSTI-based and PI-based cohorts, respectively.

### Weight and BMI Change in Females

Female INSTI initiators had a 2.5 kg (5.3 lb) greater weight gain (2.7 vs 0.2 kg; 95% CI [0.7–4.2]) compared with PI initiators (Figure 2B). Among female INSTI initiators, a higher proportion experienced ≥5% weight gain (37.5% vs 26.4%; OR=1.7; 95% CI [1.1–2.6]) or ≥10 kg (22 lb) weight gain (13.0% vs 6.0%; OR=2.3; 95% CI [1.1–2.3]; Figure 3B).

Female INSTI initiators had a 0.7 kg/m^2^ greater mean BMI increase (mean baseline BMI 30.4 kg/m^2^) compared with female PI initiators, but the difference was not statistically significant (3.5% vs 3.8%; mean difference = 0.69; 95% CI [−0.02–1.4]) (Figure 2B). A significantly higher proportion of female INSTI initiators experienced BMI increases ≥5% (37.1% vs 26.5%; OR=1.7; 95% CI [1.1–2.5]) compared with female PI initiators.

### Weight and BMI Changes in Females by Baseline BMI

Among females with baseline BMI ≥25 kg/m^2^, INSTI initiators had a 3.4 kg (7.5 lb) greater weight gain (2.4 kg [5.3 lb] vs −1.1 kg [−2.4 lb]; 95% CI [1.3–5.5]) compared with PI initiators ( Figure 2B ) and a higher proportion of INSTI initiators experienced a ≥5%, ≥10%, or extreme weight gain ( Figure 3B ). Among females with baseline BMI <25 kg/m^2^, the absolute differences in weight gain or BMI ( Figure 2B ) and the proportion of patients who experienced weight gain or BMI changes ( Figure 3B ) were not statistically significant between regimens.

## DISCUSSION

After a mean ~9.3-month follow-up, we observed a mean 1.3 kg (2.9 lb) greater weight gain among INSTI initiators versus PI initiators, with INSTI initiators having 30% greater odds of ≥5% weight gain. Based on the mean baseline weight for this study population, 5% weight gain for a typical patient would be ≥4.1 kg (9.0 lb). INSTI initiators also experienced 0.4 kg/m^2^ greater BMI increase versus PI initiators. Other outcomes showed increased weight and BMI for INSTI initiators versus PI initiators, with generally larger differences for females and overweight patients. These findings are consistent with previous studies reporting greater weight gain with INSTIs versus other regimens,^11^,^14^,^19^ and among ARV-experienced patients who switch to INSTI-based regimens.^13^

Similar differences in weight gain were reported by Bourgi et al. (dolutegravir vs raltegravir vs PI-based regimens, 6.1 vs 3.4 vs 4.1 kg)^14^ and Norwood et al. (INSTI-based vs PI-based regimen, 2.9 vs 0.7 kg),^13^ both after 18 months. Also, Bakal et al. reported larger BMI increases (INSTI-based vs PI-based regimen [1.6 vs 0.4 kg/m^2^, per year]), with higher risk among females and overweight patients.^11^ In an open-label clinical trial, Venter et al. reported greater weight gain and treatment-emergent obesity at 48 weeks for subjects taking dolutegravir/emtricitabine/TDF (3.2 kg, 7%) versus efavirenz/emtricitabine/TDF (1.7 kg, 6%), with significantly higher gain among females.^19^ In a pooled analysis of eight clinical trials, Sax et al. reported that INSTIs were associated with approximately 1.5 kg and 1.3 kg greater weight gain than PIs or NNRTIs, respectively, with female and African American patients having the greatest risk of weight gain.^16^

Notably, weight gain has been associated with an increased risk of chronic diseases such as hypertension and diabetes,^10^,^20^ which are increasingly prevalent among older PLWH,^21^ who in turn represent a growing proportion of PLWH.^4^ Approximately 50% of patients in our study were ≥50 years old at baseline, 30% had hypertension and approximately 60% were overweight, underscoring the potential importance of our findings. Furthermore, INSTIs are commonly prescribed, so even small increases in mean weight gain may have important implications for population health management, and risks regarding INSTI-related weight gain have recently been noted in guidelines for HIV treatment.^12^

Differences in weight gain between regimens may be related to “return-to-health,” a common phenomenon in PLWH.^16^,^22^ However, our cohorts were well matched on demographic/clinical variables and only 2% to 3% were underweight at baseline. Additionally, this study only included the last follow-up weight/BMI measures that were ≥90 days post-index, whereas return-to-health is most likely to occur shortly after ARV initiation. Lastly, there is no reason to expect differences between regimens related to the return-to-health phenomenon, given the high efficacy of these contemporary ARVs. Nevertheless, it would be valuable to assess weight changes over a longer follow-up time, but this would have required an earlier patient intake period, which would have limited the ability of this study to focus on currently prescribed regimens.

This study has several strengths, including a large and diverse population of real-world PLWH from multiple provider networks in the United States, with a substantial proportion having comorbidities such as hypertension, obesity, and Type II diabetes. Inclusion of a diverse population increases the external validity and generalizability of this study, although future studies should include larger numbers of high-risk patients (e.g., women, African Americans). The Optum EHR database includes data (e.g., bodyweight, BMI) that are usually absent in administrative claims data. Another strength of this study is the PS matching approach, which was designed to reduce selection biases from measured confounders and improve internal validity of estimates. Lastly, this study assessed various clinically meaningful thresholds such as proportion with weight gain ≥5%, extreme weight gain (weight gain ≥95th percentile for the overall study population), or ≥90th or 95th percentile BMI increase.

This study also has limitations. Prescription records from EHR do not necessarily indicate whether the patient received or took the medication. The first HIV diagnosis or prescription for ARV observed may not correspond to the patient’s first diagnosis, since patients may have returned to care after a gap or switched from a provider that does not provide data to the Optum EHR database. Lastly, we observed that a higher proportion of INSTI-based versus PI-based regimens included tenofovir alafenamide fumarate (TAFs) (27.8% vs 2.4%), which has been associated with weight gain, especially in combination with INSTIs.^19^ Despite use of PSM analysis, the small number of PI initiators whose regimen included TAF prevented us from determining the relative contribution of TAF versus INSTIs. However, as the ADVANCE trial demonstrated,^19^ INSTI-based regimens without TAF also lead to greater weight gain than otherwise identical PI-based regimens, suggesting that greater use of TAF by INSTI initiators is unlikely to fully account for the differences we observed. Nevertheless, further studies are warranted.

## CONCLUSIONS

Relative to patients newly initiating PI-based regimens, patients initiating INSTI-based regimens were more likely to experience weight gain within 12 months of initiation and had a greater mean weight gain. Increased weight and BMI for INSTI-based regimens were especially noteworthy among females and patients with baseline BMI ≥25 kg/m^2^. These findings may help health care providers choose optimal treatments for HIV management.

## Supplementary Information



## Figures and Tables

**Table 1:** Demographic and Clinical Characteristics

Parameter	Drug Class		*P* Value
	PI-Based Regimen (n = 794)	INSTI-Based regimen (n = 794)	
**Age, Mean (SD)**	49.9 (11.6)	48.4 (11.6)	0.015
**Age Category, n (%)**			
18–24	10 (1.3)	10 (1.3)	
25–34	89 (11.2)	100 (12.6)	
35–49	246 (31.0)	283 (35.6)	
50–64	385 (48.5)	348 (43.8)	
65+	64 (8.1)	53 (6.7)	
**Female, n (%)**	211 (26.6)	209 (26.3)	0.909
**Race, n (%)**			0.134
African American	339 (42.7)	293 (36.9)	
Asian	2 (0.3)	2 (0.3)	
Caucasian	393 (49.5)	434 (54.7)	
Other/Unknown	60 (7.6)	65 (8.2)	
**Region, n (%)**			0.560
Midwest	271 (34.1)	279 (35.1)	
South	277 (34.9)	249 (31.4)	
West	41 (5.2)	50 (6.3)	
Northeast	144 (18.1)	156 (19.6)	
Other/Unknown	61 (7.7)	60 (7.6)	
**Plan Type, n (%)**			0.001
Commercial	93 (11.7)	163 (20.5)	
Medicaid	74 (9.3)	65 (8.2)	
Medicare	69 (8.7)	55 (6.9)	
Other^a^	132 (16.6)	158 (19.9)	
Missing	426 (53.7)	353 (44.5)	
**Index Year, n (%)**			0.026
2016	454 (57.2)	410 (51.6)	
2017	269 (33.2)	285 (35.9)	
2018	71 (8.9)	99 (12.5)	
**Baseline Weight, Mean (SD)**	82.2 (20.6)	83.2 (19.9)	0.326
**Baseline BMI, Mean (SD)**	27.6 (6.9)	27.9 (6.9)	0.410
**Baseline BMI Category, n (%)**			0.247
Underweight (BMI <18.5)	18 (2.3)	25 (3.1)	
Normal (BMI 18.5–24.9)	304 (38.3)	269 (33.9)	
Overweight (BMI 25.0–29.9)	243 (30.6)	261 (32.9)	
Obese (BMI ≥30)	229 (28.8)	239 (30.1)	
**Baseline Comorbidities**			
QCI Score, Mean (SD)	3.3 (0.03)	3.4 (0.03)	0.337
**Individual Conditions, n (%)**			
Prediabetes/Glucose Intolerance	12 (1.5)	9 (1.1)	0.510
T2DM	94 (11.8)	93 (11.7)	0.938
MI	33 (4.2)	21 (2.6)	0.097
PVD	9 (1.1)	11 (1.4)	0.653
CHF	26 (3.3)	22 (2.8)	0.558
Hypertension	234 (29.5)	244 (30.7)	0.584
Hyperlipidemia	149 (18.8)	155 (19.5)	0.702
Obesity	220 (27.7)	236 (29.7)	0.375
NASH	14 (1.8)	17 (2.1)	0.586
AIDS	492 (62.0)	521 (65.6)	0.130
Cancer	56 (7.1)	51 (6.4)	0.617
**Prior Medication Use, n (%)**			
Diabetes Therapies	69 (8.7)	68 (8.6)	0.929
Psychiatric/Neurologic Therapies	130 (16.4)	152 (19.1)	0.149
Steroid Hormone	122 (15.4)	129 (16.2)	0.630
Hormone Therapy/Contraception	10 (1.3)	14 (1.8)	0.411
Appetite Stimulants/Suppressants	12 (1.5)	16 (2.0)	0.446
Antihypertensives	101 (12.7)	108 (13.6)	0.603
**Index Regimen**			
**PI Regimen Type, n (%)**			
DRV-Based^b^	409 (51.5)	N/A	N/A
ATV-Based	256 (32.2)	N/A	N/A
Other PI-Based Regimens	129 (16.2)	N/A	N/A
**PI with Booster, n (%)**^c^	739 (93.1)	N/A	N/A
**INSTI Regimen Type, n (%)**			
BIC-Based	N/A	7 (0.9)	N/A
DTG-Based^d^	N/A	349 (44.0)	N/A
EVG-Based	N/A	385 (48.5)	N/A
RAL-Based	N/A	53 (6.7)	N/A
**TAF Containing, n (%)**	19 (2.4)	221 (27.8)	N/D

Abbreviations: AIDS, acquired immunodeficiency syndrome, ATZ, atazanavir; BMI, body mass index; BIC, bictegravir; CHF, congestive heart failure; DRV, darunavir; DTG, dolutegravir; EVG, elvitegravir; INSTI, integrase strand transfer inhibitors; MI, myocardial infarction; N/A, not applicable; N/D, not determined; NASH, nonalcoholic steatohepatitis; PI, protease inhibitors; PVD, peripheral vascular disease; QCI, Quan Charlson Comorbidity Index; RAL, raltegravir; SD, standard deviation; TAF, tenofovir alafenamide fumarate; T2DM, Type II diabetes mellitus.

a Other includes: Multiple, Uninsured, Unknown, or Other.

b Patients with both DRV- and ATV-based regimens were counted under DRV-based regimen (n = 4).

c Regimen includes a booster or regimen is a single tablet regimen containing a booster.

d Patients with both DTG- and EVG-based regimens were counted under DTG-based regimen (n = 1).

**Table 2:** Index BMI Category and Proportion of Patients with BMI Category Shifts During Follow-Up

Index BMI Category	PI-Based Regimens								
	Post-Index BMI Category								
	Underweight		Normal		Overweight		Obese		Total
	n	%	n	%	n	%	n	%	n
Underweight (BMI <18.5)	8	44.4%	9	50.0%	0	0.0%	1	5.6%	18
Normal (BMI 18.5–24.9)	12	4.0%	228	75.2%	58	19.1%	5	1.7%	303
Overweight (BMI 25.0–29.9)	0	0.0%	30	12.3%	180	74.1%	33	13.6%	243
Obese (BMI ≥30)	0	0.0%	5	2.2%	32	14.0%	192	83.8%	229

Index BMI Category	INSTI-Based Regimens								
	Post-Index BMI Category								
	Underweight		Normal		Overweight		Obese		Total
	n	%	n	%	n	%	n	%	n
Underweight (BMI <18.5)	7	28.0%	17	68.0%	1	4.0%	0	0.0%	25
Normal (BMI 18.5–24.9)	8	3.0%	192	71.4%	60	22.3%	9	3.3%	269
Overweight (BMI 25.0–29.9)	0	0.0%	31	12.0%	183	70.7%	45	17.4%	259
Obese (BMI ≥30)	0	0.0%	1	0.4%	26	10.9%	212	88.7%	239

Abbreviations: BMI; body mass index; INSTI, integrase strand transfer inhibitor; PI, protease inhibitor.
