# Supplementary material for: Real-World Assessment of Weight Change in People with HIV-1 After Initiating Integrase Strand Transfer Inhibitors or Protease Inhibitors
Source: J Health Econ Outcomes Res. 2020 Jul 16;7(2):102–10. doi: 10.36469/jheor.2020.13457 (PMC7398611; doi:10.36469/jheor.2020.13457)
Supplement: Supplementary file 1 [file jheor-7-2-13457-s01.pdf]

Journal of Health Economics and Outcomes Research

Supplementary Online Material

Chen YW, Hardy H, Pericone CD, Chow W. Real-world assessment of weight change in people with HIV-1 after initiating integrase strand transfer inhibitors or protease inhibitors. *JHEOR*. 2020:7(2);102-110.
doi: 10.36469/jheor.2020.13457

Table S1. Codes for HIV Regimens
Table S2. Codes for HIV Diagnosis
Table S3. Codes for Pregnancy
Table S4. Codes for Comorbidities
Table S5. Codes for Medication Use
Table S6: Codes for Quan-Charlson Comorbidities
Table S7. Patient Attributes: Pre/Post Matching

This supplementary material has been provided by the authors to give readers additional information about their work.

This is an open-access article distributed under the terms of the Creative Commons Attribution 4.0 International License (CCBY-4.0). View this license's legal deed at http://creativecommons.org/licenses/by/4.0 and legal code at http://creativecommons.org/licenses/by/4.0/legalcode for more information.

Table S1. Codes for HIV Regimens

ARV Regimen	Drug Subclass	Trade Name	Generic Name	GPI	HCPCS
PI-based regimen[1]	PI	Reyataz	Atazanavir (ATV)	12104515%	
	PI	Prezista	Darunavir (DRV)	12104520%	
	PI	Lexiva	Fosamprenavir	12104525%	
	PI	Norvir	Ritonavir*	12104560%	
	PI	Invirase	Saquinavir	12104580%	S0140
	PI	Aptivus	Tipranavir	12104585%	
	PI	Agenerase	Amprenavir**	12104510%	
	PI	Crixivan	Indinavir**	12104530%	
	PI	Viracept	Nelfinavir**	12104545%	
	PI/PE	Evotaz	Atazanavir/Cobicistat	12109902220330	
	PI/PE	Prezcobix	Darunavir/Cobicistat	12109902270320	
	PI/PI	Kaletra	Lopinavir/Ritonavir	1210990255%	
	PI/PE/NRTI/TAF	Symtuza	Darunavir/Cobicistat/ Emtricitabine/Tenofovir AF[¥]	12109904200320	
INSTI-based regimen[2]	INSTI	Tivicay	Dolutegravir (DTG)	12103015%	
	INSTI	Isentress	Raltegravir	12103060%	
	INSTI	Vitekta	Elvitegravir (EVG)[§]	1210302000%	
	INSTI/NRTI/NRTI	Triumeq	Dolutegravir/Abacavir/Lamivudine	12109903150320	
	INSTI/NNRTI	Juluca	Dolutegravir/Rilpivirine	12109902280320	
	INSTI/PE/NRTI/NRTI	Stribild	Elvitegravir/Cobicistat/ Emtricitabine/Tenofovir DF	1210990430%	
	INSTI/NRTI/TAF	Biktarvy	Bictegravir (BIC)/ Emtricitabine/ Tenofovir AF[¥]	12109903240330	
	INSTI/PE/NRTI/TAF	Genvoya	Elvitegravir/Cobicistat/ Emtricitabine/Tenofovir AF[¥]	1210990429%	
	INSTI/NRTI	Dovato	Dolutegravir/Lamivudine	12109902260320	
NRTI-based Regimen	NRTI	Vemlidy	Tenofovir alafenamide (TAF)[¥]	12352083200320	
	NRTI/TAF	Descovy	Emtricitabine/Tenofovir AF[¥]	12109902290320	
	NRTI	Ziagen	Abacavir (ABC)	12105005%	
	NRTI	Emtriva	Emtricitabine (FTC)	12106030%	
	NRTI	Epivir	Lamivudine (3TC)	12106060%	
	NRTI	Viread	Tenofovir DF (TDF)	12108570%	
	NRTI	Retrovir	Zidovudine (ZDV)	12108085%	J3485, S0104
	NRTI	Hivid	Zalcitabine**	12106085%	
	NRTI	Zerit	Stavudine (d4T)**	12108070%	
	NRTI	Videx	Didanosine (DDI)**	12105015%	S0137
	NRTI/NRTI	Epzicom	Abacavir/Lamivudine	1210990220%	
	NRTI/NRTI/NRTI	Trizivir	Abacavir Sulfate/Lamivudine/ Zidovudine	1210990320%	
	NRTI/NRTI	Truvada	Emtricitabine/Tenofovir DF	1210990230%	
	NRTI/NRTI	Cimduo	Lamivudine/Tenofovir DF	12109902470330	
	NRTI/NRTI	Combivir	Lamivudine/Zidovudine	1210990250%	

Table S1. Codes for HIV Regimens

ARV Regimen	Drug Subclass	Trade Name	Generic Name	GPI	HCPCS
NNRTIs-based Regimen	NRTI/NNRTI/TAF	Odefsey	Emtricitabine/Rilpivirine/Tenofovir AF[¥]	12109903390320	
	NNRTI	Pifeltro	Doravirine (DTG) (approved on Aug 30, 2018)	12109025000320	
	NNRTI	Sustiva	Efavirenz (EFV)	12109030%	
	NNRTI	Intelence	Etravirine	12109035%	
	NNRTI	Viramune	Nevirapine	12109050%	
	NNRTI	Edurant	Rilpivirine	12109080100320	
	NNRTI	Rescriptor	Delavirdine**	12109020%	
	NNRTI/NRTI/NRTI	Atripla	Efavirenz/Emtricitabine/Tenofovir DF	1210990330%	
	NNRTI/NRTI/NRTI	Symfi, Symfi Lo	Efavirenz/Lamivudine/Tenofovir DF	12109903330340	
	NRTI/NNRTI/NRTI	Complera	Emtricitabine/Rilpivirine/Tenofovir DF	1210990340%	
	NNRTI/NRTI/NRTI	Delstrigo	Doravirine/Lamivudine/Tenofovir DF (approved on Aug 30, 2018)	12109903270320	
Other Regimens	Fusion inhibitor	Fuzeon	Enfuvirtide	12102530%	J1324
	CCR5 Antagonist	Selzentry	Maraviroc	12102060%	
	PE	Tybost	Cobicistat	12109530000320	
	Post-Attachment Inhibitors	Trogarzo	Ibalizaumab-uiyk (approved on March 6, 2018)	12102240302020	

Abbreviation:
CCR5: C-C chemokine receptor type 5; FDC: Fixed-dose combination; GPI: Generic Product Identifier; HCPCS: Healthcare Common Procedure Coding System; INSTI: Integrase inhibitor; NNRTI: Non-nucleoside reverse transcriptase inhibitors (NNRTIs); NRTI: Nucleoside reverse transcriptase inhibitor; PE: Pharmacokinetic enhancer; PI: Protease inhibitor; TAF: Tenofovir alafenamide;

Source:
https://aidsinfo.nih.gov/understanding-hiv-aids/fact-sheets/21/58/fda-approved-hiv-medicines/

Note:
[1] PI-based regimen: It can be single tablet regimen with PI as one of the components, or multi-tablet regimen containing at least 1 PI
[2] INSTI-based regimen: It can be single tablet regimen with INSTI as one of the components, or multi-tablet regimen containing at least 1 INSTI
[¥] Any regimen containing tenofovir AF was flagged as a "TAF-containing regimen", in addition to being categorized as PI-based, INSTI-based, NRTI-based, NNRTI-based, or Other
* Although ritonavir is a PI, it is generally used as a pharmacokinetic enhancer as recommended in the Guidelines for the Use of Antiretroviral Agents in Adults and Adolescents Living with HIV and the Guidelines for the Use of Antiretroviral Agents in Pediatric HIV Infection.
** Drugs are no longer available and/or recommended for use in the US by the HHS HIV/AIDS medical practice guidelines. There drugs may still be used in fixed-dose combination formulations
[§] Vitekta (standalone elvitegravir) was voluntarily withdrawn from market by Gilead in February 2017 due to low use (<50 patients in US)

Table S2. Codes for HIV Diagnosis

Diagnosis	ICD-9 code	ICD-10 code
HIV-1	042.%, 795.71, V08.%	B20.%, R75.%, Z21.%
HIV-2	079.53	B97.35%

Abbreviation:
HIV: Human Immunodeficiency Virus; ICD: International Classification of Diseases

Table S3. Codes for Pregnancy

Diagnosis	ICD-9 code	ICD-10 code	CPT code
Pregnancy	V22.%-V24.%, V27.%, V28.%, V30%-V39%, 640.%-677.%, 760.%-763.%	Z34.%-Z39.%, O%, P00.%-P03.%	59000-59899

Abbreviation:
CPT: Current Procedural Terminology; HIV: Human Immunodeficiency Virus; ICD: International Classification of Diseases

Table S4. Codes for Comorbidities

Diagnosis	ICD-9 code	ICD-10 code
Pre-Diabetes	790.29	R73.03
Type 2 Diabetes	250.% (fifth digit with 0 or 2 only)	E11.%
Myocardial infarction	410.%, 412.%	I21.%, I22.%, I25.2%
Peripheral vascular disease	440.2%, 440.3%, 440.4%, 440.8%, 440.9%, 443.22, 443.81, 443.89, 443.9, 444.22, 444.81, 445.02	E08.5%, E10.5%, E11.5%, E13.5%, I70.2%, I70.3%, I70.4%, I70.5%, I70.6%, I70.7%, I70.8%, I70.9%, I73.89, I73.9%, I79.8%, I74.3%, I74.5%, I75.029
Congestive heart failure	402.01, 402.11, 402.91, 404.91, 404.93, 391.8%, 398.91, 392.0%, 428.%	I01.8%, I09.81, I02.0%, I50.%
Hypertension	401.% - 405.%	I10.% - I16.%
Hyperlipidemia	272.0%, 272.1%, 272.2%, 272.3%, 272.4%	E78.0%, E78.1%, E78.2%, E78.3%, E78.4%, E78.5%
Obesity	278.00, 278.01, 278.03, V85.3%, V85.4%	E66.0%-E66.2%, E66.8%, E66.9%, Z68.3%, Z68.4%
Nonalcoholic steatohepatitis/non-alcoholic fatty liver	571.8% (used before 10/1/2015)	K75.81 (NASH), K76.0 (non-alcoholic fatty liver)
AIDS	042.%	B20.%
Cancer	140.% – 172.%, 174.% – 195.8%, 200.% – 208.%	C00.%–C26.%, C30.%–C34.%, C37.%–C41.%, C43.%, C45.%–C58.%, C60.%–C76.%, C81.%–C85.%, C88.%, C90.%

Abbreviation:
AIDS: Acquired Immunodeficiency Syndrome; ICD: International Classification of Diseases; NASH: Non-alcoholic Steatohepatitis

Table S5. Codes for Medication Use

Medication	GPI	HCPCS
**Diabetes therapies**		
Insulin	2710%, 279910%	J1815, J1817, E0784, S5550-S5571, G9147, S9353
Thiazolidinediones	2760%, 279940%, 279978%, 279980%	
Sulfonylureas	2720%, 279970%, 279978%	
Biguanides/Meglitinides	2725%, 279925%, 279960%, 279970%, 279980%, 279988%, 279990%, 2728%, 279950%	
DPP-4 inhibitors	2755%, 279925%, 279930%, 279940%, 279965%	
GLP-1 Receptor Agonists	2717%, 279910%	
Alpha-Glucosidase inhibitors	27.5	
**Psychiatric/Neurologic therapies**		
Tricyclic antidepressants	58.2	
Selective Serotonin Reuptake Inhibitors	58.16	
Antipsychotics	5907%, 5910%, 5915%, 5916%, 5920%, 5925%, 5930%, 5940%, 5950%	
Antiseizure/Anticonvulsants	72500010%, 72500020%, 72500030%, 72600020%, 72600030%, 72600075%, 72600090%, 72600040%	
Others (bupropion, nefazodone, lithium, mirtazapine)	58300040%, 58120050%, 59500010%, 58030050%	Others (bupropion, nefazodone, lithium, mirtazapine)
Tricyclic antidepressants	58.2	Tricyclic antidepressants
Steroid Hormones	22%, 8915%	J0702, J7624, J7622, J7633, J7627, J7634, J7626, J8540, J1094, J1100, J7312, J7637, J7638, J1700, J1710, J1720, J1020, J1030, J1040, J2920, J2930, J7509, J2650, J7510, J7506, J7512, J3300, J3301, J3302, J3303, J7683, J7684
Hormone therapy/Contraception (estrogen, testosterone, progesterone, tesamorelin)	2400%, 2499%, 231000%, 260000%, 30150085%	
Appetite Stimulants/Suppressants	61200070%, 72600075%, 61400020%, 61109902%, 21404020%, 23200040%, 50300030%	
Anti-hypertensives	36%	J0210, J2760, J0360, S0139, J1730, J2670

Antiseizure/anticonvulsants are valproic acid, carbamazepine, gabapentin, topiramate, zonisamide, and lamotrigine.

Appetite stimulants/suppressants are phentermine, topiramate, methylphenidate, amphetamine/dextroamphetamine, megestrol, oxandrolone, dronabinol.

Abbreviations:
DPP-4: Dipeptidyl-peptidase 4; GLP-1: Glucagon-like Peptide 1; GPI: Generic Product Identifier; HCPCS: The Healthcare Common Procedure Coding System

Table S6. Codes for Quan-Charlson Comorbidities

Comorbidities	ICD-9-CM codes*	ICD-10-CM codes*	Quan 2010 weight**
Myocardial infarction	410.%, 412.%	I21.%, I22.%, I25.2%	0
Congestive heart failure	398.91, 402.01, 402.11, 402.91, 404.01, 404.03, 404.11, 404.13, 404.91, 404.93, 425.4%-425.9%, 428.%	I09.9%, I11.0%, I13.0%, I13.2%, I25.5%, I42.0%, I42.5%–I42.9%, I43.%, I50.%, P29.0%	2
Peripheral vascular disease	093.0%, 437.3%, 440.%, 441.%, 443.1%-443.9%, 447.1% 557.1%, 557.9%, V43.4%	I70.%, I71.%, I73.1%, I73.8%, I73.9%, I77.1%, I79.0%, I79.2%, K55.1%, K55.8%, K55.9%, Z95.8%, Z95.9%	0
Cerebrovascular disease	362.34, 430.% – 438.%	G45.%, G46.%, H34.0%, I60.%–I69.%	0
Dementia	290.%, 294.1%, 331.2%	F00.%–F03.%, F05.1, G30.%, G31.1%	2
Chronic Pulmonary Disease	416.8%, 416.9%, 490.%-505.%, 506.4%, 508.1%, 508.8%	I27.8%, I27.9%, J40.%–J47.%, J60.%–J67.%, J68.4%, J70.1%, J70.3%	1
Connective tissue/rheumatic disease	446.5%, 710.0% – 710.4%, 714.0% – 714.2%, 714.8%, 725.%	M05.%, M06.%, M31.5%, M32.%–M34.%, M35.1%, M35.3%, M36.0%	1
Peptic ulcer disease	531.% – 534.%	K25.%–K28.%	0
Mild liver disease	070.22, 070.23, 070.32, 070.33, 070.44, 070.54, 070.6%, 070.9%, 570.%, 571.%, 573.3%, 573.4%, 573.8%, 573.9%, V42.7%	B18.%, K70.0%–K70.3%, K70.9%, K71.3%–K71.5%, K71.7%, K73.%, K74.%, K76.0%, K76.2%–K76.4%, K76.8%, K76.9%, Z94.4%	2
Diabetes without chronic complications/mild to moderate	250.0% – 250.3%; 250.8%, 250.9%	E10.0%, E10.1%, E10.6%, E10.8%, E10.9%, E11.0%, E11.1%, E11.6%, E11.8%, E11.9%, E12.0%, E12.1%, E12.6%, E12.8%, E12.9%, E13.0%, E13.1%, E13.6%, E13.8%, E13.9%, E14.0%, E14.1%, E14.6%, E14.8%, E14.9%	0
Paraplegia and hemiplegia	334.1%, 342.%, 343.%, 344.0%-344.6%, 344.9%	G04.1%, G11.4%, G80.1%, G80.2%, G81.%, G82.%, G83.0%–G83.4%, G83.9%	2
Renal disease	403.01, 403.11, 403.91, 404.02, 404.03, 404.12, 404.13, 404.92, 404.93, 582.0% – 583.7%, 585.%, 586.%, 588.0%, V42.0%, V45.1%, V56.%	I12.0%, I13.1%, N03.2%–N03.7%, N05.2%–N05.7%, N18.%, N19.%, N25.0%, Z49.0%–Z49.2%, Z94.0%, Z99.2%	1
Diabetes with chronic complications	250.4% – 250.7%	E10.2%–E10.5%, E10.7%, E11.2%–E11.5%, E11.7%, E12.2%–E12.5%, E12.7%, E13.2%–E13.5%, E13.7%, E14.2%–E14.5%, E14.7%	1
Any malignancy, including lymphoma and leukemia, except malignant neoplasm of skin	140.% – 172.%, 174.% – 195.8%, 200.% – 208.%, 238.6%	C00.%–C26.%, C30.%–C34.%, C37.%–C41.%, C43.%, C45.%–C58.%, C60.%–C76.%, C81.%–C85.%, C88.%, C90.%–C97.%	2
Moderate or severe liver disease	456.0%-456.2%, 572.2%-572.8%	I85.0%, I85.9%, I86.4%, I98.2%, K70.4%, K71.1%, K72.1%, K72.9%, K76.5%, K76.6%, K76.7%	4
Metastatic solid tumor	196.% – 199.%	C77.%–C80.%	6
AIDS/HIV	042.%, 043.%, 044.%	B20.%–B22.%, B24.%	4

Abbreviations:
AIDS: Acquired Immunodeficiency Syndrome; HIV: Human Immunodeficiency Virus; ICD-9/10-CM: International Classification of Diseases 9th/10th revision Clinical Modification

Table S7. Patient Attributes: Pre/Post Matching

Matching Criteria	Prior to Matching						Post Matching					
	PI-base regimen		INSTI-based regimen		*|d*|*	*p-value*	PI-based regimen		INSTI-based regimen		*|d*|*	*p-value*
**Number of patients**	811		4306				794		794			
**Age, n (%)**												
18-24	10	1.2%	240	5.6%	24.1%	<0.0001	10	1.3%	10	1.3%	0.0%	0.501
25-34	92	11.3%	811	18.8%	21.0%		89	11.2%	100	12.6%	4.3%	
35-44	130	16.0%	857	19.9%	10.1%		128	16.1%	150	18.9%	7.3%	
45-54	304	37.5%	1222	28.4%	19.5%		296	37.3%	273	34.4%	6.0%	
55-64	210	25.9%	899	20.9%	11.9%		207	26.1%	208	26.2%	0.3%	
65+	65	8.0%	277	6.4%	6.1%		64	8.1%	53	6.7%	5.3%	
**Female, n (%)**	216	26.6%	996	23.1%	8.1%	0.031	211	26.6%	209	26.3%	0.6%	0.909
**Region, n (%)**												
Midwest	273	33.7%	1549	36.0%	4.9%	0.116	271	34.1%	279	35.1%	2.1%	0.560
South	284	35.0%	1308	30.4%	9.9%		277	34.9%	249	31.4%	7.5%	
West	42	5.2%	213	4.9%	1.1%		41	5.2%	50	6.3%	4.9%	
Northeast	151	18.6%	878	20.4%	4.5%		144	18.1%	156	19.6%	3.9%	
Other/Unknown	61	7.5%	358	8.3%	2.9%		61	7.7%	60	7.6%	0.5%	
**Race, n (%)**												
African American	345	42.5%	1613	37.5%	10.4%	0.011	339	42.7%	293	36.9%	11.9%	0.134
Asian	2	0.2%	29	0.7%	6.3%		2	0.3%	2	0.3%	0.0%	
Caucasian	404	49.8%	2250	52.3%	4.9%		393	49.5%	434	54.7%	10.4%	
Other/Unknown	60	7.4%	414	9.6%	8.0%		60	7.6%	65	8.2%	2.3%	
**Index year, n (%)**												
2016	465	57.3%	1956	45.4%	24.0%	<0.0001	454	57.2%	410	51.6%	11.1%	0.026
2017	272	33.5%	1675	38.9%	11.2%		269	33.9%	285	35.9%	4.2%	
2018	74	9.1%	675	15.7%	20.0%		71	8.9%	99	12.5%	11.4%	
**Baseline weight, mean (SD)**	82.26	20.51	82.57	20.48	1.5%	0.691	82.23	20.63	83.23	19.91	4.9%	0.326
**Baseline BMI, mean (SD)**	27.57	6.87	27.47	6.75	1.5%	0.704	27.57	6.87	27.85	6.87	4.1%	0.410
**Comorbid condition, n (%)**												
Prediabetes/glucose intolerance	12	1.5%	108	2.5%	7.4%	0.076	12	1.5%	9	1.1%	3.3%	0.510
T2DM	94	11.6%	432	10.0%	5.0%	0.180	94	11.8%	93	11.7%	0.4%	0.938
MI	33	4.1%	123	2.9%	6.6%	0.065	33	4.2%	21	2.6%	8.3%	0.097
PVD	9	1.1%	68	1.6%	4.1%	0.314	9	1.1%	11	1.4%	2.3%	0.653
CHF	26	3.2%	116	2.7%	3.0%	0.416	26	3.3%	22	2.8%	2.9%	0.558

Table S7. Patient Attributes: Pre/Post Matching

Matching Criteria	Prior to Matching						Post Matching					
	PI-base regimen		INSTI-based regimen		|*d**|	*p-value*	PI-based regimen		INSTI-based regimen		|*d**|	*p-value*
Hypertension	236	29.1%	1149	26.7%	5.4%	0.155	234	29.5%	244	30.7%	2.7%	0.584
Hyperlipidemia	151	18.6%	813	18.9%	0.7%	0.861	149	18.8%	155	19.5%	1.9%	0.702
Obesity	220	27.1%	1156	26.8%	0.6%	0.869	220	27.7%	236	29.7%	4.5%	0.297
Nonalcoholic steatohepatitis	14	1.7%	76	1.8%	0.3%	0.939	14	1.8%	17	2.1%	2.7%	0.586
AIDS	499	61.5%	2912	67.6%	12.8%	0.001	492	62.0%	521	65.6%	7.6%	0.130
Cancer	56	6.9%	294	6.8%	0.3%	0.936	56	7.1%	51	6.4%	2.5%	0.617
**Baseline medication use, n (%)**												
Diabetes therapies	70	8.6%	348	8.1%	2.0%	0.600	69	8.7%	68	8.6%	0.4%	0.929
Psychiatric/neurologic therapies	131	16.2%	808	18.8%	6.9%	0.078	130	16.4%	152	19.1%	7.3%	0.149
Steroid hormone	123	15.2%	783	18.2%	8.1%	0.039	122	15.4%	129	16.2%	2.4%	0.630
Hormone therapy/contraception	10	1.2%	93	2.2%	7.2%	0.085	10	1.3%	14	1.8%	4.1%	0.411
Appetite stimulants/suppressants	12	1.5%	120	2.8%	9.1%	0.031	12	1.5%	16	2.0%	3.8%	0.446
Anti-hypertensives	103	12.7%	552	12.8%	0.4%	0.926	101	12.7%	108	13.6%	2.6%	0.603

Note:
*d: standard difference, with |d| ≥10% being considered as significant.

Abbreviations:
AIDS: Acquired Immunodeficiency Syndrome; BMI: Body Mass Index; CHF: Congestive Heart Failure; INSTI: Integrase Strand Inhibitor; MI: Myocardial Infarction; PI: Protease Inhibitor; PVD: Peripheral Vascular Disease; SD: Standard Deviation; T2DM: Type 2 Diabetes Mellitus
